# Hypoxia and hypoxia-inducible factor signals regulate the development, metabolism, and function of B cells

**DOI:** 10.3389/fimmu.2022.967576

**Published:** 2022-08-15

**Authors:** Jinwei Zhang, Xiaoqian Wu, Jideng Ma, Keren Long, Jing Sun, Mingzhou Li, Liangpeng Ge

**Affiliations:** ^1^ Chongqing Academy of Animal Sciences, Chongqing, China; ^2^ Key Laboratory of Pig Industry Sciences, Ministry of Agriculture, Chongqing, China; ^3^ Chongqing Camab Biotech Ltd., Chongqing, China; ^4^ Farm Animal Genetic Resource Exploration and Innovation Key Laboratory of Sichuan Province, Sichuan Agricultural University, Chengdu, China

**Keywords:** hypoxia, hypoxia-inducible factor signaling, B cell biology, development, metabolism, function

## Abstract

Hypoxia is a common hallmark of healthy tissues in physiological states or chronically inflamed tissues in pathological states. Mammalian cells sense and adapt to hypoxia mainly through hypoxia-inducible factor (HIF) signaling. Many studies have shown that hypoxia and HIF signaling play an important regulatory role in development and function of innate immune cells and T cells, but their role in B cell biology is still controversial. B cells experience a complex life cycle (including hematopoietic stem cells, pro-B cells, pre-B cells, immature B cells, mature naïve B cells, activated B cells, plasma cells, and memory B cells), and the partial pressure of oxygen (PO_2_) in the corresponding developmental niche of stage-specific B cells is highly dynamic, which suggests that hypoxia and HIF signaling may play an indispensable role in B cell biology. Based on the fact that hypoxia niches exist in the B cell life cycle, this review focuses on recent discoveries about how hypoxia and HIF signaling regulate the development, metabolism, and function of B cells, to facilitate a deep understanding of the role of hypoxia in B cell-mediated adaptive immunity and to provide novel strategies for vaccine adjuvant research and the treatment of immunity-related or infectious diseases.

## Introduction

The two types of host defense systems are innate and adaptive immunity. They work together to resist the invasion of foreign pathogens and maintain the homeostasis of internal environments, and are crucial for the evolutionary fitness of mammals. Innate immunity can quickly block and eliminate nonspecific pathogens and provides the first line of defense against infectious challenges (1). Adaptive immunity develops more slowly and not only destroys specific pathogens through the production of high-affinity, class-switched antibodies, but also creates immunological memory for similar antigens (2). Effective immunity in healthy individuals occurs at focal sites of immune activity, known as physiological immune niches, including bone marrow (BM), spleen, lymph nodes, and the intestinal mucosa (3, 4). In contrast, pathological immunological niches are sites of tissue pathology associated with inflammation and tissue dysfunction (5, 6). Microenvironmental features of immunological niches play a key regulatory role in the development, metabolism, and function of resident immune cells (7).

The vast majority of immune cells originate in the BM and migrate to peripheral immune tissues through the circulation for further maturation and effector function development. Immunological niches are microenvironments with dynamic partial pressure of oxygen (PO_2_), which induce different degrees of hypoxia adaptation by activating cellular hypoxia-inducible factor (HIF) signaling (8). Hypoxia and HIF signaling play an important role in the development and function of innate immune cells and T cells, but their effects on B cell biology remains controversial (9–11). B cells have a complex life cycle that is driven by both intrinsic (genetic programs) and extrinsic (antigen and cytokines) cues (12). The variable PO_2_ at corresponding immunological niches of stage-specific B cells is likely to be closely interlinked with the functional requirements of stage-specific cell adaptation (13). Given that pathological immunological niches have been expertly reviewed elsewhere (7), this review summarizes recent advances concerning how hypoxia and HIF signaling regulate the development, metabolism, and function of B cells in physiological immunological niches.

## B cell life cycle

B cells were first discovered by Cooper in 1965, and three lineages have been identified to date, including B1, B2, and regulatory B cells (Bregs) (14, 15). B1 cells (including B1a and B1b), which originate from the fetal liver and are located in the peritoneum, spleen, and mucosa (16). B2 cells are produced in the BM throughout life and represent the bulk of the B cell pool, and contain follicular B (FOB) and marginal zone B (MZB) cells (17). Bregs represent a class of B cells with immunosuppressive properties and play a key role in the establishment of B cell tolerance primarily through the production of the anti-inflammatory cytokine IL-10 (18). This review focuses principally on the B2 lineage unless otherwise stated.

**Figure 1:**
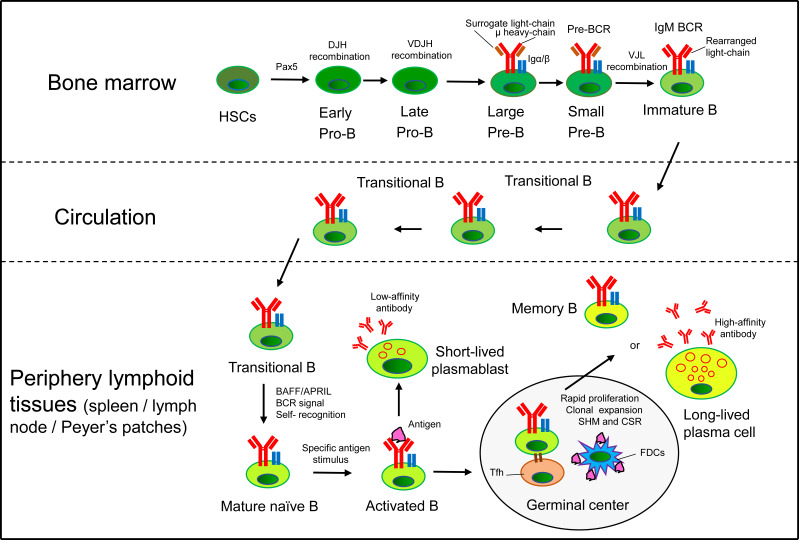
B cell life cycle. In the BM, HSCs commit to the B cell lineage producing pro-B cells. Pro-B cells undergo VDJ recombination at the Ig heavy-chain locus to differentiate into pre-B cells, which rearrange Ig light-chain genes to further develop into IgM^+^ immature B cells. Immature B cells egress from the BM and migrate into peripheral lymphoid tissues *via* the circulation (transitional B cells) to complete maturation. Mature naïve B cells respond to Td antigens and develop into short-lived plasmablasts that secrete low-affinity antibodies. Activated B cells extensively proliferate with the help of Tfh cells in the GC of periphery lymphoid tissues (such as spleen, lymph node and Peyer’s patches), where B cells undergo SHM and CSR. Finally, B cells differentiate into high-affinity antibody-secreting plasma cells and long-lived memory B cells to provide specific and long-term protection for the body.

B2 cells originate from multipotent hematopoietic stem cells (HSCs) in the BM, where common lymphoid progenitors (CLP) are committed to pro-B cells through the expression of the transcription factor Pax5 (19). Pro-B cells undergo V(D)J recombination at the immunoglobulin (Ig) heavy-chain locus to differentiate into pre-B cells, which rearrange Ig light-chain genes to further develop into IgM^+^ immature B cells. Immature B cells undergo B cell receptor (BCR) editing and produce a diverse BCR repertoire with limited self-antigen recognition (20, 21). Then, they enter the circulation as transitional B cells, and migrate into peripheral lymphoid tissues to complete maturation, finally differentiating into FOB or MZB (22–24). FOB recirculate and reside in the periphery lymphoid follicles, while MZB locate to the margins of follicles, and respond to T cell-dependent (Td) and T cell-independent (Ti) antigens, respectively (6). Mature naïve B cells undergo class-switch recombination (CSR) and develop into short-lived plasmablasts that secrete low-affinity antibodies. Activated B cells extensively proliferate with the help of T follicular helper (Tfh) cells in the germinal center (GC), where B cells undergo BCR diversification by introducing random mutations into the V(D)J fragments of the heavy- and light-chain genes (known as somatic hypermutation, SHM) (25). Then, B cells differentiate into high-affinity antibody-secreting plasma cells and long-lived memory B cells to provide specific and long-term protection **(** **Figure 1** **)**. B cell life cycle are precisely regulated through several defining transcription factors that have been systematically reviewed elsewhere (17, 26).

## HIF signaling

Hypoxic responses, whether physiological [embryogenesis (27)] or pathological [cancer (28)], external [organism level (29)] or internal [cellular level (30)], have been a research hotspot in recent decades. All metazoan cells have evolved an oxygen-sensing system that enables rapid adaptation to fluctuations in the PO_2_ of their resident niches. The master regulator of this system is the family of HIFs, a kind of heterodimeric transcription factors that comprise an O_2_-sensitive α subunit (HIF-1α/-2α/-3α) and a constitutively expressed β subunit [Aryl hydrocarbon receptor nuclear translocator 1/2 (ARNT1/2)] (30). HIF-1α is ubiquitously expressed and HIF-2α shows a more tissue-specific expression pattern (such as heart, lung, kidney, and placenta) and is also expressed in some immune cell subtypes (macrophages, neutrophils, and lymphocytes), whereas HIF-1α and HIF-2α have distinct but overlapping target gene sets (31, 32). Additionally, HIF-3α has been reported to be expressed only by epithelial cells in the lung and kidney and have both positive and negative effects on hypoxia-modulated gene expression depending on the specific context (33, 34).

**Figure 2:**
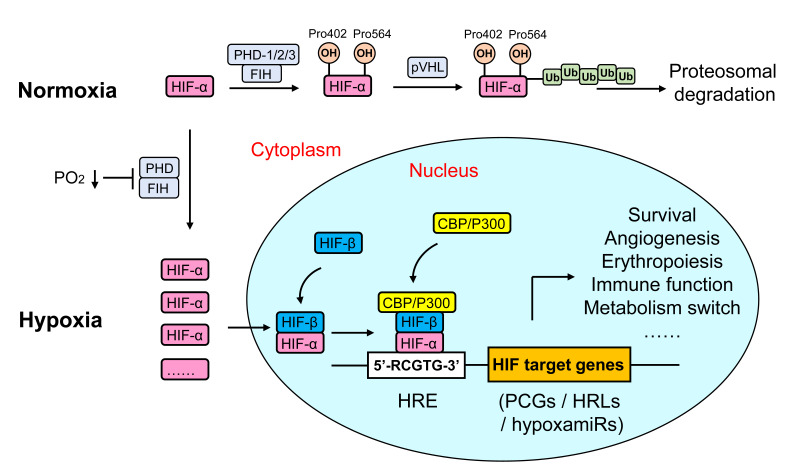
Regulation of HIF signaling in metazoan cells. HIFs are a kind of heterodimeric transcription factors that comprise an α subunit (O_2_-sensitive) and a β subunit (constitutive). Under normoxia, proline residues in the ODD of HIF-α are hydroxylated by PHDs or FIH, which facilitates HIF-α binding to the VHL E3 ubiquitin ligase complex, and leads to polyubiquitination and proteosomal degradation. Hypoxia induces HIF-α protein stabilization by inactivating PHD and FIH. Activated HIF-α translocates from the cytoplasm to the nucleus, where it dimerizes with HIF-1β and recruits CBP/p300, and then binds to HRE (5’-RCGTG-3’) in the promoter or enhancer of HIF-regulated genes [including PCGs, hypoxia-responsive lncRNAs (HRLs) and hypoxamiRs], finally regulate a variety of hypoxia-adaptive pathways such as angiogenesis, anaerobic metabolism, and erythropoiesis.

HIF-α is composed of a Per-ARNT-Sim (PAS) domain and an N-terminal basic-helix-loop-helix (bHLH) domain that mediates dimerization and transcriptional activation, respectively. Under normoxia, two conserved proline residues (Pro402 and Pro564) in the O_2_-dependent degradation domain (ODD) of HIF-α are hydroxylated by one of three prolyl hydroxylase domain enzymes (PHD), which facilitates HIF-α binding to the von Hippel-Lindau (VHL) E3 ubiquitin ligase complex and leads to polyubiquitination and proteosomal degradation (35). Moreover, HIF-α is also hydroxylated on a specific asparagine residue (Asn803) in the C-terminal transactivation domain (CTAD) by factor-inhibiting HIF-1 (FIH-1), which hinders the transcriptional activation of HIF through inhibition of the binding of HIF to co-activator CREB-binding protein (CBP)/p300 (36). Hypoxia induces HIF-α protein stabilization by inactivating PHD and FIH. Activated HIF-α translocates from the cytoplasm to the nucleus, where it dimerizes with HIF-1β and recruits CBP/p300, and then binds to hypoxia response element (5’-RCGTG-3’) in the promoter or enhancer of HIF-regulated genes, of which more than 300 protein coding genes (PCGs) have been reported (37, 38). Moreover, hypoxia also regulates gene expression at the posttranscriptional level by modulating a specific subset of miRNAs [hypoxamiRs (39)] and lncRNAs [hypoxia responsive lncRNAs, HRLs (40)]. The consequence of the cellular hypoxia response activates multiple hypoxia-adaptive pathways involved in angiogenesis, anaerobic metabolism, and erythropoiesis, which triggers a cellular metabolism shift toward glycolysis and reestablishes O_2_ supply (8, 41) **(** **Figure 2** **)**.

## Hypoxia niches in the B cell life cycle

The vasculature supplies O_2_ and nutrients for all organs, tissues and cells of the body depending on their metabolic needs, meanwhile disposing of waste such as CO_2_ and metabolic products to establish a balance between supply and consumption (42, 43). Whether a tissue is hypoxic is determined by a combination of factors, including vessel size and density, distance from cells to the nearest vessel, cell density, and metabolic demands (6). The differences in these factors make the various tissues of the body form different hypoxic landscapes.

Under physiological conditions, PO_2_ varies throughout the body in a descending order from arterial blood (~13%), main organs (~6%; e.g., brain, liver, lung) to lymphoid tissues (~3%; e.g., spleen). The development process from hematopoietic progenitors to immature B cells occurs in the BM where the presence of hypoxic niches (average PO_2_ 2.25%) and a PO_2_ gradient has been elucidated from direct [two-photon phosphorescence lifetime microscopy (44)] and indirect evidence [proteomic analysis (45), HIF-1α and its regulated genes (46) and surrogate hypoxic marker staining (47)]. Different degrees of hypoxic niches [e.g., severe hypoxia in perisinusoidal regions (1.3%) and lower hypoxia in endosteal regions (2.9%) (44)] in the BM have an important effect on cell fate and function, including self-renewal and quiescence maintenance in stem cells, B cell lineage recruitment, and V(D)J rearrangement in pro-B/pre-B cells (48). Nevertheless, the PO_2_ landscape of B cells at different developmental stages in BM needs to be accurately matched and further studied. Immature B cells enter into well-oxygenated circulation (average PO_2_ 13.2%) and are in a relatively normoxic state before transport to various secondary lymphoid tissues. Then, B cells reencounter hypoxic niches within lymphoid tissues (average PO_2_: spleen 2.3% and lymph node 1.85%) and interact with specific antigens to differentiate into plasma cells and memory cells. Of note, the majority of GCs (PO_2_ approximately 1%) within secondary lymphoid tissues are poorly vascularized or are >40 μm away from blood vessels and highly express HIF protein, which is essential for the formation of antibody repertoire diversity and the development of an effective immune response (49–51). It is noteworthy that activated B cells mainly experience hypoxia in the light zone of GC, but the precise value of PO_2_ in the light/dark zone of GCs has not been directly reported (51). Overall, the B cell life cycle experiences hypoxia niches over a wide range of oxygen tensions (heterogeneity of local PO_2_) and HIF-α is activated to varying degrees, which will broadly shape B cell fate and profoundly affect B cell metabolism and function (**Table 1**).

**Table 1:** Characteristics of B cells at different developmental stages (6, 44, 46, 49, 51–58).

B cell life cycle	Location	PO_2_	Detection methods	Landmark event/function	Markers
HSCs	BM	~2.25% [intravascular: ~2.7% (1.5-4.2%) Extravascular: ~1.8% (0.6-2.8%)]	Pimonidazole (hypoxyprobe); Two-photon pO_2_ microscope	Stemness and self-renewal	CD34, CD117, KIT, IL7R, ATXN1, FLT3
Pro-B				Heavy-chain recombination	CD34, CD38, CD24, CD19, CD45, CD43, RAG1/2, EBF1, PAX5, SOX4, LEF1, IGLL1, TdT, CD179B, Vpreb, CD10, IL7R
Pre-B				Light-chain recombination	CD34, CD38, CD24, CD19, CD45, CD43, CD79B, IL7R, IGH, RAG1/2, SOX4, LEF1, EIF4EBP1, PAX5, Vpreb, RSP27
Immature B				BCR editing, BAFF/APRIL	CD19, CD20, CD45, CD38, CD24, EIF4EBP1, EBF1, TNFRSF13C
Transitional B	Circulation	13.20%	Oxygen sensor	Transition-state B cell from central to peripheral	CD20, CD38, CD24, IGHM, EBF1, PAX5
Mature naïve B	Periphery lymphoid tissue	Spleen: ~2.30% (0.5-4.5%) Lymph node: ~1.85% (0.1-3.6%) Peyer’s patches:? Germinal center:~1%	Pimonidazole (hypoxyprobe); Oxygen microelectrode	BCR signal, Self-antigen recognition	CD19, CD20, CD24, CD79A, EIF4EBP1, PAX5, TNFRSF13C, IGHM, Vpreb
Activated B				Specific antigen response, SHM, CSR	CD19, MS4A1, LTB, CRIP1, S100A10, S100A4, CD79A, JCHAIN, IGHM, IGHA, IGHG
Plasma cell				Specific antibody production	IGHM, IGKC/IGLC2, CD20, CD79A, TNFRSF13C
Memory B				Immune memory formation	CD11c, AICDA, CD20, CD27, IGHM

## Hypoxic regulation of B cell biology

The B cell life cycle frequently encounters hypoxia in physiological states, which leads to the variable activation of HIF in immunological niches. The temporal, spatial, and dynamic nature of hypoxic stimuli induces a series of precise regulatory events in B cells biology. We next discuss how hypoxia and HIF signaling regulate the development, metabolism, and function of B cells in physiological immunological niches.

### Hypoxia regulates B cell development

Many studies have shown that hypoxia plays a vital role in regulating B cell development, mainly through the HIF signaling pathway. The most direct evidence is that HIF-1α gene deficiency causes abnormal B cell development and autoimmunity (autoantibodies accumulation) (59–61). From the perspective of different immunological niches, hypoxic niches in BM play an important role in the maintenance of HSCs homeostasis and in the regulation of cellular survival, proliferation, and differentiation (62). HIF signaling activation by PHD inhibitors promotes HSCs quiescence (reduces proliferation and enhances myeloid potential) in an HIF-dependent manner and facilitates hematopoiesis and improves blood recovery (including B cells) after severe irradiation (63). Hypoxia also plays an important synergistic effect on improving antibody-secreting plasma cell survival by regulating paracrine survival factors like fibronectin and YWHAZ (also known as 14-3-3zeta/delta) from BM stroma cells (64). Chabi et al. reported that hypoxia specifically favors *in vitro* human lymphoid development from early hematopoietic progenitor cells and enhances the *in vivo* B cell potential of lymphoid-primed multipotent progenitors (48). B cell lymphopoiesis occurs primarily in the BM and consists of multiple cellular stages. Florian et al. found that HIF activity is high in pro-B and pre-B cells and decreases in immature B cells. Stage-specific HIF suppression is required for normal B cell development because constitutive HIF-1α activation (VHL deficiency) leads to immature B cell developmental arrest and a reduction in peripheral B cell numbers (52).

In peripheral immunological niches, hypoxia causes an HIF-1α-dependent growth arrest in mouse splenic B cells through upregulation of the expression of the cyclin-dependent kinase inhibitors p21 and p27 (65). Hypoxia regulates WiL2-NS B cell (a human spleen-derived B lymphoblast cells) viability by mediating CXCR4 expression through HIF-1α/Nrf2 cooperatively binding to its promoter (66). Matthieu et al. demonstrated that hypoxia increases the generation of plasmablasts starting from peripheral blood-derived memory B cells by increasing cell cycle and division number *via* HIF-1α and HIF-2α (50).

The GC is a specialized micro-anatomy structure that develops in peripheral lymphoid tissues, where activated B cells undergo SHM and CSR to produce high-affinity, class-switched antibodies. The GC consists of a dark zone and light zone, where the dark zone contains actively dividing B cells known as centroblasts and the light zone contains nondividing B cells known as centrocytes (67). Sung et al. reported that activated B cells experience hypoxia predominantly in the GC light zone of the spleen, lymph node, and Peyer’s patches. B lymphoblasts rapidly proliferate in the dark zone, but they reduce proliferation and increase B cell death in the light zone (51). Therefore, hypoxia regulates B cell development in central and peripheral immunological niches from multiple aspects, mainly mediated by the HIF signaling pathway.

Hypoxic preconditioning is often used clinically as a protocol that induces a naturally protective immunophenotype in some diseases like strokes (68), this is at least partially effected *via* B cell function. Nancy et al. reported that repetitive hypoxic preconditioning (RHP) can enhance the protection from stroke-induced injury, mechanistically, as RHP increases in CD1d^hi^CD5^+^ regulatory B cells and inhibits the proliferation, development, and differentiation of B cells and B-T cell adaptive immune interactions in the spleen (69). Moreover, HIF activation or knockdown in other immune cells affect the development of B cells after vaccine immunization. Sung et al. found that the frequency of GC-phenotype B cells (GL7^+^ CD95^+^ IgD^neg^ B220^+^) and antigen-specific B cells were substantially reduced in HIF knockdown mice after immunization with hapten-conjugated ovalbumin, which indicates that HIF plays a vital role in GC immune responses and the production of specific antibody-secreted plasma cells (70).

### Hypoxia regulates B cell metabolism

From the antigen-independent B cell development in the BM to the antigen-driven production of antibody-secreted plasma cells in the peripheral GC, life for a B cell experiences dynamic metabolic processes to support the demands of cell differentiation and effector function (71).

B cells undergo multiple rounds of metabolic state changes during their development in the BM. Dynamic hypoxia in the BM activates HIF signaling, thereby affecting the metabolic activity of B cells at different developmental stages, which is necessary for the development and functional improvement of B cells (72). HSCs preferentially utilize glycolysis instead of mitochondrial oxidative phosphorylation for their energy needs (73). Early pro-B cells have a high metabolic demand to meet their own rapid proliferation needs, so both glycolysis and oxidative phosphorylation (OXPHOS) are increased because of the action of IL-7 signaling (74). After completing VDJ recombination in of heavy chains, late pro-B cells reduce their metabolic activity and gradually become quiescent state. Early-stage large pre-B cells express a surface pre-BCR formed from IgM pairs with a surrogate light chain, which triggers rapid proliferation and clonal expansion accompanied by increased glycolysis and OXPHOS (75). Large pre-B cells undergo a proliferative burst with the highest glycolysis activity occurring in B cell progenitors in the BM. Rapid proliferation at this stage increases cellular oxygen consumption to further enhance microenvironmental hypoxia and HIF-1α stability. Then, this activates the expression of glucose transporters and glycolytic enzymes to maintain high levels of glycolysis (76). As large pre-B cells differentiate into nonproliferating small pre-B cells, the metabolic activity of these cells gradually declines but rises again when these cells complete light-chain recombination (75). After differentiating into immature B cells, the metabolism levels of these cells decreases and may be dependent on fatty acids as their primary metabolic substrates (74). B cell development in the BM was shown to be highly dependent on glucose or glycolysis, and HIF-1α-deficient B cells in the BM are less capable of using glucose because glucose transporters and glycolytic-enzyme expression have been greatly decreased. HIF-1α-enabled glycolysis is required in a developmental stage-specific manner during B cell development in the BM (75). Additionally, some cytokines or growth factors, such as IGF-1 (77) and hematopoietic cell growth factors (78), are capable of regulating B cell glucose utilization and glycolysis to influence B cell fate. Studies have shown that the stromal cell-secreted cytokine IL-7 induces the PI3K-Akt pathway and promotes PLCγ-mediated mTOR activation, meaning these will contribute to promoting glucose utilization and B cell development (79, 80). Whether hypoxia directly affects these cytokines or growth factors to regulate B cell metabolism and fate remains to be further explored.

To date, relatively few studies have examined the metabolic state of transitional B cells in the circulation. Farmer et al. reported that transitional B cells had a relatively high metabolic activity, which adapted to the high oxygen levels in the circulation. The induction of metabolic quiescence was shown to be required for the switch from transitional B cells to FOB cells (22). In peripheral lymphoid tissues, naive mature B cells are in a quiescent state with low metabolic activity (81). When mature naive B cells respond to antigen, BCR stimulation mediates a rapid increase in glycolysis and OXPHOS (82). The BAFF-BAFF receptor signaling pathway induces glucose utilization through inhibiting glycogen synthase kinase 3 (Gsk3) through the PI3K-Akt pathway. Gsk3 is an important metabolic checkpoint regulator that promotes the survival of naive recirculating B cells and maintains metabolic quiescence (83). Activated B cells increase the expression of HIF-1α and cMyc to maintain glycolysis. Subsequently, B cells enter the GC cycle between the dark zone and the light zone. HIF-1α was shown to be dynamically regulated in GC B cells to ensure the different functions of HIF-1α at different stages (67). B cells in dark zones differentiate into centroblasts accompanied by BCL-6 upregulation and cMyc inhibition, and HIF-1α-mediated glycolytic activity under normoxia (aerobic glycolysis) provides energy for rapid proliferation of centroblasts (84). B cells then enter into the GC light zone and differentiation into centrocytes. Oxygen- and nutrient-poor microenvironments in the light zone maintain HIF-1α and AMPK expression and promote cell catabolism. cMyc is then upregulated in centrocytes and induces a glycolytic burst (anaerobic glycolysis) to meet the metabolic demands of B cells from the cells in the light zone reentering the dark zone. Centroblasts and centrocytes upregulate the expression of LDHB and MCT1 to utilize extracellular lactate (83). A recent study, however, demonstrated that highly proliferative B cells in the GC possess a special metabolic profile in which they mainly rely on fatty acid oxidation rather than on glycolysis. This view contrasts with previous reports that GC B cells rely on glycolysis for energy, which may be related to the different cell types used in the experiments (activated B cells *in vitro* and GC B cells *ex vivo* were used, respectively) (85). B cells differentiate into memory B cells or plasma cells after GC reaction. Memory B cells self-renew slowly and are relatively metabolically quiescent. Although plasma cells are highly terminally differentiated cells with no or weak proliferation abilities, they have high metabolic activity (high glucose uptake and glycolysis/OXPHOS) to supply energy for the production and secretion of specific antibodies (86). The effect of hypoxia on plasma cell metabolism remains to be further studied.

### Hypoxia regulates B cell function

The ultimate mission of B cells in vertebrates is to produce high-affinity antibody-secreted plasma cells and long-lived memory B cells to eliminate specific pathogens and provide long-term protection (87). Many studies have demonstrated that hypoxia and HIF signaling can regulate B cell function through diverse and sophisticated manners, such as B cell migration, immune responses, SHM, and CSR. A nuanced understanding of the role of HIF in B cell function is very important, because the HIF signaling pathway is pharmacologically tractable and may serve as an important target for vaccine adjuvants and immunity-related treatment (6).

B cell migration between different immunological niches, such as BM, circulation, and peripheral immune tissues, is essential for their normal function. Hypoxia has been reported to have different effects on B cell migration depending on B cell location. Hypoxia upregulates the expression of CXCR4 at both the transcriptional and posttranslational levels (88), which induces the migration of peripheral blood B cells while inhibiting that of GC B cells, thus increasing mature B cell egress from Peyer’s patches and promoting the retention of immature B cells in the BM (89, 90). HIF also can modulate the B cell immune response, but the specific role of HIF in antibody formation is still controversial. Burrows et al. demonstrated that HIF-1α activation reduces BCR editing and repertoire diversity in murine B cells (52). Cho et al. found that hypoxia in the GC light zone has intricate effects on Td B cell response. Specifically, in this context, HIF activation reduces GC B cell proliferation, impairs affinity maturation, decreases the pro-inflammatory IgG2c isotype, and weaken secondary antibody responses. HIF hyperactivation is also detrimental to B cell function, but physiological hypoxia in normal GC may be time-specific and of functional benefit (51). In contrast, Abbott et al. reported that hypoxic conditions *in vitro* accelerate Ig CSR and plasma cell formation, while respiratory hyperoxia suppresses CSR and the GC reaction during immunization *in vivo (* 49). Although these two studies appear to have opposite results, they imply that precise and appropriate HIF activation is necessary for an optimal immune response, and a targeted and timely manipulation of the HIF signaling pathway may provide an efficient approach for immune intervention.

SHM introduces high-frequency point mutations (10^-4^–10^-3^/base pair/cell division) in the variable regions of the Ig heavy and light chains higher than the rate of genome-wide spontaneous mutations (10–9), which facilitates iterative screening to obtain high-affinity antibodies (91). CSR events allow antibody-switching from IgM to IgG, IgA, or IgE isotypes by C-region exchange to achieve diverse effect functions. Hypoxia has been reported to affect affinity maturation and class-switching by modulating SHM and CSR, respectively, yet a detailed mechanism remains to be elucidated (6). Some DNA damage repair-related enzymes, such as activation-induced deaminase (AID), play an important role in the CSR and SHM (92). Hypoxia impairs antibody class-switching to the IgG2c isotype by decreasing AID expression at the mRNA and protein levels, but it does not reduce IgA switching (51). Reportedly, however, hypoxic conditions (1% O_2_) *in vitro* also can accelerate plasma cell formation and IgM^+^→IgG1^+^ class-switching (49). The contrasting effects of hypoxia in CSR may be associated with the specific differences in B cell subsets, as well as the extent and duration of hypoxia (6). Hypoxia has been reported to inhibit mismatch repair proteins (MSH2/MSH6) and base-excision repair proteins in the tumor-derived hypoxic niches, but promotes error-prone translation DNA synthesis (TLS) polymerase and induce mutagenesis, which suggested that hypoxia may affect SHM and CSR by mediating DNA-repair proteins (93). Besides SHM and CSR, RAG1/RAG2-mediated V(D)J gene rearrangement is a key factor in the formation of antibody repertoire diversity (94). The role of hypoxia in V(D)J gene rearrangement warrants further exploration in future studies, because this may provide an important target for the development of vaccine adjuvants in specific scenarios.

Noncoding RNAs, such as miRNA (95), lncRNA (96) and circRNA (97), play an important regulatory role in B cell development and function at the post-transcriptional level (98). For example, miR-181b impairs CSR by negatively regulating AID in B cells (99) and miR-155 promotes GC formation *in vivo* and the generation of IgG1-secreting plasma cells by downregulating the target gene *Pu.1* (100). Hypoxia also has been reported to affect B cell function in part through regulating noncoding RNAs. A typical example is miR-210, a master hypoxamiR induced by HIF-1α and Oct-2 during B cell activation, which causes abnormalities in B cell subsets and functions, including B cell proliferation and CSR (101). Establishing the detailed regulatory network of hypoxamiR and hypoxia-responsive lncRNAs in B cell lymphogenesis is beneficial for systematically elucidating the effect of hypoxia on B cell biology.

Moreover, hypoxia has an effect on other immune cell interactions by modulating some cytokines (such as IL-2, IL-4, IL-10, and IFN-γ) to indirectly regulate B cell function (102). HIF-1α depletion from CD4^+^ T cells reduces the frequencies of antigen-specific GC B cells and overall antigen-specific antibodies after NP-ovalbumin immunization (70). Conversely, hypoxia also affects T cell immune responses (CD8^+^ T cell) by promoting the secretion of B cell-derived extracellular vesicles through HIF-1α-mediated Rab27a transcription (103). Intriguingly, HIF signaling is also activated in an oxygen-independent manner. The downstream activation of BCR, toll-like receptor (TLR) and cytokine receptor signaling cascades stabilizes HIF-α protein in B cells, which highlights the important role of oxygen-independent HIF signaling in B cell immune responses (6, 13).

## Concluding remarks

B cells have a complex life cycle, and the oxygen tensions in corresponding developmental niches of stage-specific B cells are highly dynamic. PO_2_ fluctuation in different niches affects B cells lymphogenesis mainly through the HIF signaling pathway. Many studies from different perspectives have shown that hypoxia plays an important regulatory role in the development, metabolism, and function of B cells. It is necessary to recognize that hypoxia is a double-edged sword for B cells—that is, hypoxia in physiological states is required for B cell biology, whereas acute or severe hypoxia may induce or exacerbate B cell dysfunction. The regulation of hypoxia on B cells is sophisticated and multifaceted, such as HIF-dependent or HIF-independent pathways, regulation of protein-coding genes or through noncoding RNAs at the post-transcriptional level in a regulatory manner, and by direct regulation of B cells or indirect regulation of other immune cells as regulatory objects.

It will be interesting to elucidate when HIF is activated in the B cell life cycle, how hypoxia plays a regulatory role in different B cell resident immune niches, and what the regulatory outputs are in different scenarios. A comprehensive understanding of the complex regulatory mechanisms of hypoxia on B cell biology will (a) facilitate precise therapeutic manipulation of the HIF signaling pathway [B cell stage-specific or niche-specific HIF regulator (activator or inhibitor)] to modulate immune responses during vaccination as well as some immunity-related diseases (especially autoimmune diseases); (b) be beneficial to comprehensively evaluate the effect of drug treatment or vaccine immunity from the perspective of intrinsic hypoxia in the body’s internal environment; (c) help to compare the similarities and differences between physiological hypoxia and tumor’s microenvironmental hypoxia, and provide theoretical support for improving the effectiveness of antitumor therapy.

The following aspects deserve further research in future studies. (a) The direct detection method of PO_2_ in smaller micro-structure like light/dark zone of GCs is to be developed or optimized, based on this, the PO_2_ at corresponding niches of stage-specific B cells remains to be precisely decrypted. (b) Like HIF-1α, HIF-2α also plays a pivotal role in other immune cells, and the expression pattern and function of HIF-2α in B cells remains unclear (8). (c) Additionally, Bregs are immunosuppressive cells that support immunological tolerance, all while exerting immunosuppressive effects by secreting specific cytokines, such as IL-10, IL-35, and TGF-β (15). To our knowledge, the regulatory effects of hypoxia on Bregs have been rarely reported thus far. (d) Furthermore, the gastrointestinal mucosa is an important immunological niche, in which physiological hypoxia exists and can modulate innate immunity by promoting epithelial cell barrier functioning and regulating resident immune cells (104). How hypoxia affects B cell development and SIgA (a secretory antibody that play a critical role in mucosal immunity) production in intestinal mucosal immune tissues (such as Peyer’s patches) needs further study, which is beneficial to the development of therapeutic targets for intestinal-related diseases. (e) Last but not least, the SARS-CoV-2 virus, an important threat to global public health, has been reported to cause systemic hypoxia and a notable reduction in B cell numbers. Hypoxia contributes to the pronounced and persistent B cell pathology of acute SARS-CoV-2 (105). Evaluating the effect of early oxygen therapy on B cell immunodeficiency provides a reference for the treatment of similar major infectious diseases (106, 107).

## Author contributions

Conceptualization, JZ and LG. Literature investigation, XW, JM, KL, and JS. Writing original draft: JZ and XW. Supervision: ML and LG. All authors have read and agreed to the published version of the manuscript.

## Funding

This work was funded by the National Natural Science Foundation of China (32072687), the Natural Science Foundation of Chongqing (cstc2021jcyj-msxmX0630), the Performance Incentive and Guidance Project for Scientific Research Institutions in Chongqing (20524), the Special funded project of Chongqing Postdoctoral Fund (21310), Sichuan International Science and Technology Innovation Cooperation/Hong Kong, Macao and Taiwan Science, Technology Innovation Cooperation Project (2021YFH0033) and Science Foundation of the Sichuan Province (2022YFQ0022).

## Conflict of interest

Authors JZ and LG were employed by the company Chongqing Camab Biotech Ltd.

The remaining authors declare that the research was conducted in the absence of any commercial or financial relationships that could be construed as a potential conflict of interest.

## Publisher’s note

All claims expressed in this article are solely those of the authors and do not necessarily represent those of their affiliated organizations, or those of the publisher, the editors and the reviewers. Any product that may be evaluated in this article, or claim that may be made by its manufacturer, is not guaranteed or endorsed by the publisher.
